# Isolation and characterization of two novel groups of kanamycin-resistance ColE1-like plasmids in *Salmonella enterica* serotypes from food animals

**DOI:** 10.1371/journal.pone.0193435

**Published:** 2018-03-07

**Authors:** Chin-Yi Chen, Terence P. Strobaugh, Ly-Huong T. Nguyen, Melanie Abley, Rebecca L. Lindsey, Charlene R. Jackson

**Affiliations:** 1 Molecular Characterization of Foodborne Pathogens Research Unit, Eastern Regional Research Center, Agricultural Research Service, U.S. Department of Agriculture, Wyndmoor, Pennsylvania, United States of America; 2 Bacterial Epidemiology and Antimicrobial Resistance Research Unit, Richard B. Russell Agricultural Research Center, Agricultural Research Service, U.S. Department of Agriculture, Athens, Georgia, United States of America; Cornell University, UNITED STATES

## Abstract

While antimicrobial resistance in *Salmonella enterica* is mainly attributed to large plasmids, small plasmids may also harbor antimicrobial resistance genes. Previously, three major groups of ColE1-like plasmids conferring kanamycin-resistance (Kan^R^) in various *S*. *enterica* serotypes from diagnostic samples of human or animals were reported. In this study, over 200 Kan^R^ *S*. *enterica* isolates from slaughter samples, collected in 2010 and 2011 as a part of the animal arm of the National Antimicrobial Resistance Monitoring System, were screened for the presence of ColE1-like plasmids. Twenty-three Kan^R^ ColE1-like plasmids were successfully isolated. Restriction fragment mapping revealed five major plasmid groups with subgroups, including two new groups, X (n = 3) and Y/Y2/Y3 (n = 4), in addition to the previously identified groups A (n = 7), B (n = 6), and C/C3 (n = 3). Nearly 75% of the plasmid-carrying isolates were from turkey and included all the isolates carrying X and Y plasmids. All group X plasmids were from serotype Hadar. Serotype Senftenberg carried all the group Y plasmids and one group B plasmid. All Typhimurium isolates (n = 4) carried group A plasmids, while Newport isolates (n = 3) each carried a different plasmid group (A, B, or C). The presence of the selection bias in the NARMS strain collection prevents interpretation of findings at the population level. However, this study demonstrated that Kan^R^ ColE1-like plasmids are widely distributed among different *S*. *enterica* serotypes in the NARMS isolates and may play a role in dissemination of antimicrobial resistance genes.

## Introduction

In the U.S., the Centers for Disease Control and Prevention estimated 48 million new episodes of foodborne illness, ~128,000 hospitalizations, and 3000 deaths each year [1]. *Salmonella enterica* serovars are among the leading bacterial pathogens, causing 11% of illnesses, 35% of the hospitalizations, and 28% of deaths [2]. Multi-drug resistant (MDR; resistance to 3 or more antibiotics) isolates pose a greater threat to human and animal health due to treatment failure, and thus are a serious food security, food safety, and public health concern [3]. The prevalence of MDR strains of *S*. *enterica* from food animals has been consistent or steadily increasing since testing began in 1997, with the exception of 2011, based on the National Antimicrobial Resistance Monitoring System (NARMS) reports [4–7]. Between 2010 and 2011, the most prevalent *S*. *enterica* serotypes isolated at slaughter from chicken, turkey, cattle, and swine were Kentucky, Hadar, Montevideo, and Derby (2010)/Adelaide (2011), respectively, [7,8].

The majority of the antimicrobial resistance studies have been focused on chromosomal cassettes/integrons [9,10] or large conjugative plasmids, such as IncA/C and IncI [11–15]. PCR typing kits have been developed for characterizing the large plasmid replicons [16,17]. Very few studies have been devoted to small plasmids although the low molecular weight plasmids were estimated to be present in about 10% of *S*. *enterica* field strains [18]. These small plasmids may carry genes encoding bacteriocins (ColE plasmids), restriction-modification systems, reverse transcriptase, the O-antigen, and antimicrobial resistance [18–22]. Thus far, small ColE1-like plasmids have been shown to carry resistance genes against kanamycin (*aph*) [19,23–26], quinolones (*qnr*) [27,28], and extended spectrum β-lactams (*bla*_CMY_) [29]. The ColE1-like plasmids utilize RNA I/II and Rom for plasmid replication/maintenance functions [30], and most also carry mobilization gene(s) that, although not self-conjugative, allow plasmid transfer with the help of other co-resident conjugative plasmids such as F and IncP [31].

Previously we characterized three major small ColE1-like plasmid groups conferring Kan^R^ (encoded by *aph(3’)-I* gene) in various *S*. *enterica* serovars of human clinical isolates and NARMS animal diagnostic samples from 2005 [23,25]. These plasmids shared moderately high levels of identity (85–92%) to each other in the RNA I/II- *rom* region [24,26]; some also carried IS elements [26]. Different groups of plasmids showed some correlation with certain serotypes [25]. To determine if these plasmids are also present in *S*. *enterica* from other sources, small plasmid groups conferring Kan^R^ from *S*. *enterica* isolated from healthy food animals were characterized. In this study, kanamycin resistant *S*. *enterica* isolates collected between 2010 and 2011 as part of NARMS were screened to investigate the occurrence and distribution of Kan^R^ ColE1-like plasmids using PCR-typing with ColE1 primers [25].

## Materials and methods

### *Salmonella* NARMS strain collection and characterization

Kan^R^ *S*. *enterica* isolates (n = 223) from slaughter samples collected by the animal arm of NARMS between 2010 and 2011 [32] were selected for screening of ColE1-like plasmids (S1 Table). All *S*. *enterica* strains were stored at -80°C in LB Lennox (Hardy Diagnostics, Santa Maria, CA) with 15% glycerol or stored at room temperature on tryptic soy agar (Hardy Diagnostics, Santa Maria, CA) slants until use.

Serotyping, antimicrobial resistance profile, and pulsed-field gel electrophoresis (PFGE) analysis were performed per NARMS criteria as previously described [14,33]. Briefly, all isolates were tested for susceptibility to 15 antimicrobials as defined by the NARMS program [32] using a semi-automated broth microdilution system (Sensititre, Trek Diagnostic Systems, Inc.). Each isolate was classified as resistant, susceptible, or intermediate using the Clinical and Laboratory Standards Institute (CLSI; formerly National Committee for Clinical Laboratory Standards) breakpoints when available; otherwise, breakpoint interpretations as defined by NARMS were used [32,34,35]. PFGE (*Xba*I) and cluster analysis was conducted as previously described [14] at the USDA VetNet Laboratory (Athens, GA) using the PulseNet 24-h *Salmonella* PFGE protocol [36]. BioNumerics software program (Applied Maths Scientific Software Development, Saint-Martens-Latem, Belgium) was applied for cluster analysis using the Dice coefficient and the unweighted pair-group method (UPGMA).

### ColE1-like replicon and *aph* gene screening

Whole-cell lysates from all Kan^R^ isolates were prepared by inoculating 200 μl of sterile water with a single isolated bacterial colony and heating at 95°C for 10 min, followed by 5 min incubation on ice. The lysates were then centrifuged at 10,000 ×g for 3 min, and the supernatants were transferred to fresh tubes. The presence of the ColE1-like replicon and the kanamycin-resistance gene, *aph3’-I*, were sequentially tested by PCR using ColE1 typing primers CC7059F and CC7062R, and APH-F1 and APH-R1 primers, respectively (S2 Table) [25]. Primers were synthesized by Integrated DNA Technologies, Inc. (Coralville, IA). Reactions were prepared in a 20 μl final volume with 1.5 μl of 10-fold diluted lysates or 0.5 μl 10-fold diluted genomic DNA prep (see below), 1× TopTaq PCR Buffer, 0.5 μM of each primer, 0.2 mM each of dNTP, 1× Q-solution and 1 U of TopTaq DNA polymerase (Qiagen). PCR samples were initially incubated for 3 min at 94°C, followed by 35 cycles of 30 sec at 94°C/ 60 sec at 57°C/ 60 sec at 72°C, and 10 min at 72°C. Primer pairs CC7059F/CC7062R and APH-F1/APH-R1 generated 351-bp and 814-bp products, respectively. An isolate was presumed to carry a Kan^R^ ColE1-like plasmid if both amplicons of the expected size were observed. Total genomic DNA from the 66 presumptive ColE1-plasmid (+) *S*. *enterica* isolates was purified using Gentra Puregene Yeast/Bacteria kit (Qiagen) and used for further characterization.

### Plasmid purification, classification, and sequencing

Total DNA (1.5 μl) from ColE1-positive *S*. *enterica* strains was used for transformation of *Escherichia coli* NEB5-alpha high efficiency competent cells (New England BioLabs, Ipswich, MA) and colonies were selected on LB agar supplemented with 50 μg/ml kanamycin A (Sigma-Aldrich, St. Louis, MO). Plasmids were prepared from up to six transformed Kan^R^ *E*. *coli* colonies using the QIAprep Spin Miniprep kit. Candidate Kan^R^ ColE1-like plasmids were categorized by restriction digests using the following enzymes: *Bgl* II, *Eco*R V, *Hin*d III, *Nco* I, *Nde* I, *Nla* III, *Pvu* I, *Pvu* II, *Sal* I, *Sca* I, *Sma* I/*Xma* I, *Spe* I, *Xba* I, and *Xho* I singularly or in combination. Approximately 100 ng of DNA was digested with *Nla* III for 2 h at 37°C and separated on 1.8% agarose 3:1 (ISC BioExpress, Kaysville, UT) containing 1:10,000 GelRed (Biotium Inc., Hayward, CA) in 1× Tris-acetate-EDTA (TAE) buffer, 160 volt-h. All restriction enzymes were purchased from New England BioLabs and digestions followed the manufacturer’s recommendation.

The *aph(3’)*-I genes and RNAI/II regions were sequenced from the PCR products or the plasmid DNA, respectively, using custom primers and the BigDye Terminator v3.1 on an ABI 3730 sequence analyzer (Life Technologies) following the manufacturer’s recommendations. Nucleotide sequences were assembled using the Sequencher program (Gene Codes, Ann Arbor, MI). Sequence similarity was compared to GenBank and custom databases using the BLAST programs [37,38]; pairwise or multiple sequence alignment was performed using Geneious (v. 6.1; Biomatters, Ltd., Aukland, NZ).

### NCBI GenBank accession numbers

RNA II region between primers CC7059F and CC7062R of the X and Y plasmid groups (represented by pX-Kan and pY2-Kan) were deposited in NCBI GenBank under accession numbers KX863344 and KX863345, respectively.

## Results

### Kan^R^ ColE1-like plasmid screen

From the 2010 and 2011 NARMS studies, a total of 223 *S*. *enterica* isolates (resistance ranging from to 2 to 11 antibiotics tested) were resistant to kanamycin, consisting of 30 serotypes. Serotypes Dublin, Heidelberg, Typhimurium, Senftenberg, and Schwarzengrund were the top five serotypes, representing 41.3%, 16.1%, 8.5%, 4.9%, and 3.1% of the Kan^R^ isolates; 14 serotypes were only represented by one isolate each. The composition of the animal source was as follows: 113 (50.6%) from cattle (cattle, ground beef, boneless beef), 68 (30.5%) from turkey (ground turkey, turkey carcass, turkey burger), 27 (12.1%) from chicken (young chicken, ground chicken), and 15 (6.7%) from swine (market hog). Among the 223 Kan^R^ isolates screened, 66 (29.6%) tested ColE1(+) by PCR, 57 (25.6%) were both ColE1(+) and Aph(+).

**Fig 1:**
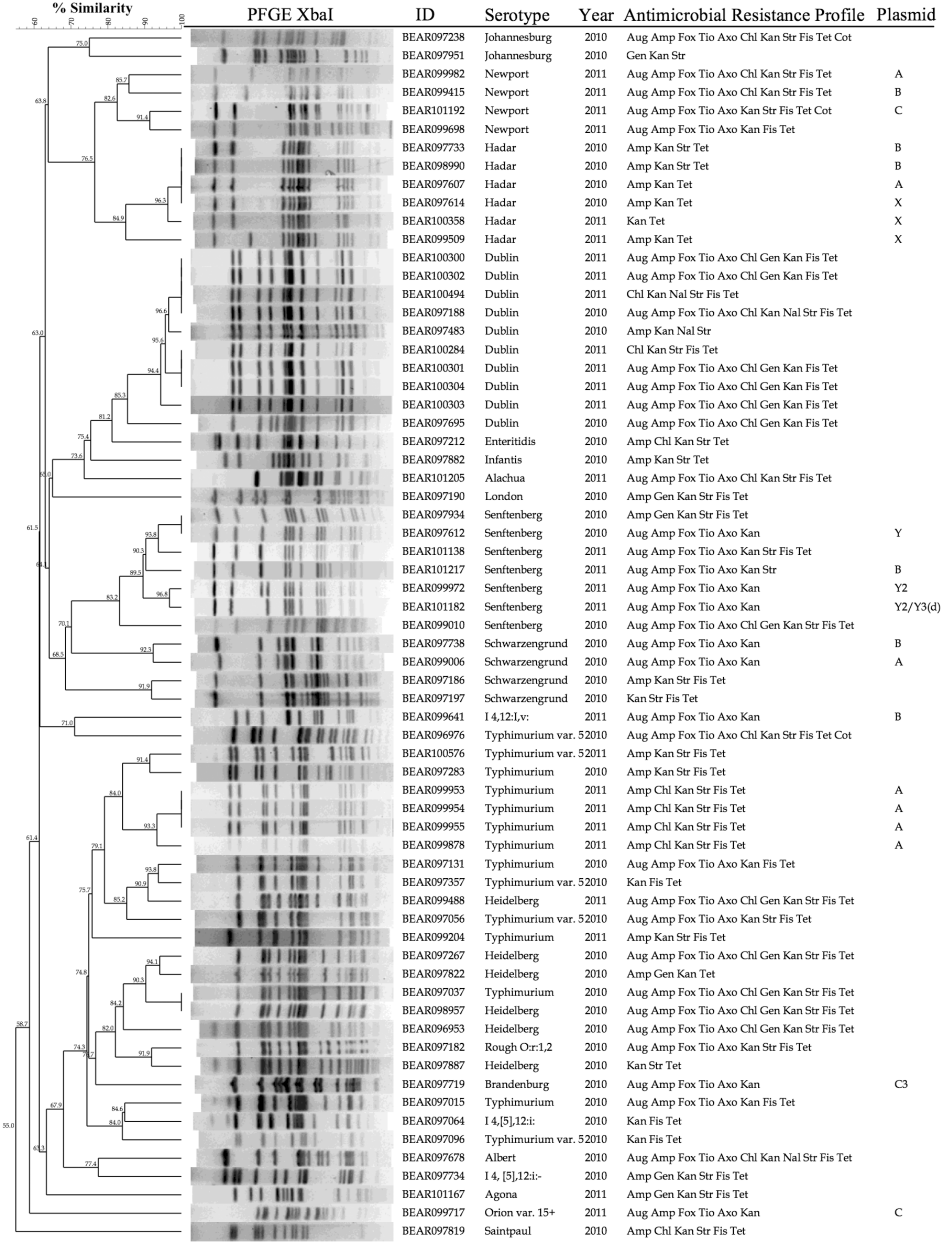
PFGE-based dendrogram, serotype, antibiotic resistance profile, and Kan^R^ ColE1-like plasmid group of the ColE(+) isolates. The dendrogram is based on PFGE analyses using BioNumerics software. Abbreviation for antimicrobial resistance phenotype: ampicillin (Amp), amoxicillin-clavulanic acid (Aug), cefoxitin (Fox), ceftiofur (Tio), ceftriaxone (Axo), chloramphenicol (Chl), gentamicin (Gen), kanamycin (Kan), nalidixic acid (Nal), sulfisoxazole (Fis), streptomycin (Str), tetracycline (Tet), Trimethoprim/sulfamethoxazole (Cot). Kan^R^ ColE1-like plasmid group is indicated in the “Plasmid” column.

DNA from 46 of the 66 ColE1(+) strains was able to transform NEB5-alpha to Kan^R^. Most of the transformations resulted in over five hundred colonies following the standard procedure; however, some DNA preps only resulted in one to a few dozen transformants. Up to six colonies were selected from each transformation experiment, and plasmids were purified for further analyses. Small high-copy-number plasmids were isolated from 22 original *S*. *enterica* strains, correlating well with the high efficiency of transformation by DNA obtained from these strains. Genomic DNA from the remaining strains resulted in fewer than four colonies from each transformation. Plasmid minipreps purified from the transformants of 10 strains resulted in smears by restriction digestions and the undigested plasmid prep did not reveal a low molecular-weight (MW) band, suggesting that high MW plasmids carrying Kan^R^ genes may be involved. These 24 strains were thus excluded from further study. A PFGE-based dendrogram generated from the 66 ColE(+) strains is shown in Fig 1.

All 22 *S*. *enterica* strains carrying Kan^R^ ColE1-like plasmids were isolated from turkey or cattle, and none from swine or chicken (Table 1). None of the Dublin and Heidelberg strains were found to carry Kan^R^ ColE1-like plasmids using this procedure. All six Kan^R^ Hadar strains carried Kan^R^ ColE1-like plasmids.

**Table 1:** *S*. *enterica* isolates carrying kanamycin-resistance ColE1-like plasmids: Serotype, antibiotic resistance profile, year of isolation and Kan^R^ ColE1-like plasmid group.

ID	Serotype	Product class	Antimicrobial resistance profile^a^	Year	Kan^R^ ColE1-like plasmid
BEAR097607	Hadar	Ground Turkey	Amp Kan Tet	2010	**A**
BEAR097612	Senftenberg	Ground Turkey	Aug Amp Fox Tio Axo Kan	2010	**Y**
BEAR097614	Hadar	Ground Turkey	Amp Kan Tet	2010	**X**
BEAR097719	Brandenburg	Turkey Carcass	Aug Amp Fox Tio Axo Kan	2010	**C3**
BEAR097733	Hadar	Turkey Carcass	Amp Kan Str Tet	2010	**B**
BEAR097738	Schwarzengrund	Turkey Carcass	Aug Amp Fox Tio Axo Kan	2010	**B**
BEAR098990	Hadar	Turkey Carcass	Amp Kan Str Tet	2010	**B**
BEAR099006	Schwarzengrund	Ground Turkey	Aug Amp Fox Tio Axo Kan	2010	**A**
BEAR099415	Newport	Ground Beef	Aug Amp Fox Tio Axo Chl Kan Str Fis Tet	2011	**B**
BEAR099509	Hadar	Ground Turkey	Amp Kan Tet	2011	**X**
BEAR099641	I 4,12:l,v:	Ground Turkey	Aug Amp Fox Tio Axo Kan	2011	**B**
BEAR099717	Orion var. 15+	Ground Turkey	Aug Amp Fox Tio Axo Kan	2011	**C**
BEAR099878	Typhimurium	Ground Beef	Amp Chl Kan Str Fis Tet	2011	**A**
BEAR099953	Typhimurium	Ground Beef	Amp Chl Kan Str Fis Tet	2011	**A**
BEAR099954	Typhimurium	Ground Beef	Amp Chl Kan Str Fis Tet	2011	**A**
BEAR099955	Typhimurium	Ground Beef	Amp Chl Kan Str Fis Tet	2011	**A**
BEAR099972	Senftenberg	Ground Turkey	Aug Amp Fox Tio Axo Kan	2011	**Y2**
BEAR099982	Newport	Ground Beef	Aug Amp Fox Tio Axo Chl Kan Str Fis Tet	2011	**A**
BEAR100358	Hadar	Turkey Carcass	Kan Tet	2011	**X**
BEAR101182	Senftenberg	Ground Turkey	Aug Amp Fox Tio Axo Kan	2011	**Y2/Y3**
BEAR101192	Newport	Ground Turkey	Aug Amp Fox Tio Axo Kan Str Fis Tet Cot	2011	**C**
BEAR101217	Senftenberg	Ground Turkey	Aug Amp Fox Tio Axo Kan Str	2011	**B**

^a^Abbreviations: ampicillin (Amp), amoxicillin-clavulanic acid (Aug), cefoxitin (Fox), ceftiofur (Tio), ceftriaxone (Axo), chloramphenicol (Chl), kanamycin (Kan), sulfisoxazole (Fis), streptomycin (Str), tetracycline (Tet), Trimethoprim/sulfamethoxazole (Cot)

### Classification of the Kan^R^ ColE1-like plasmids

**Fig 2:**
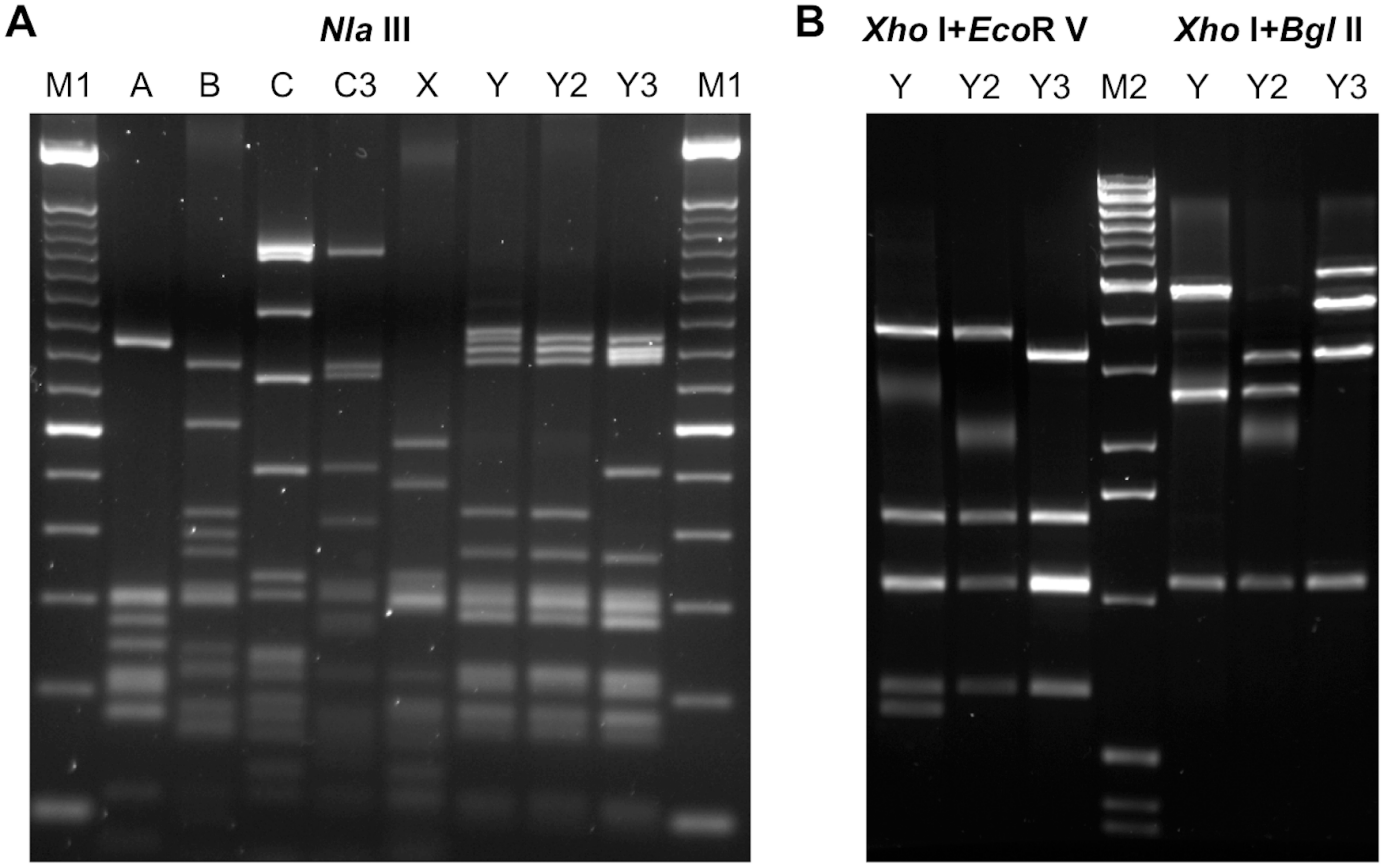
Restriction digests of representative Kan^R^ ColE1-like plasmids. A. *Nla* III digests of representative plasmid groups: A (from BEAR099953), B (from BEAR097733), C (from BEAR101192), C3 (from BEAR097719), X (from BEAR099509), Y (from BEAR097612), Y2 (from BEAR099972) and Y3 (from BEAR101182). M1, 100 bp ladder (Invitrogen). B. *Xho* I+*Eco*R V and *Xho* I+*Bgl* II digests of plasmid groups X, Y, Y2, and Y3; M2, 1kb Extension ladder (Invitrogen).

Based on the restriction pattern analyses using approximately 12 enzyme combinations, we identified two new plasmid groups: X (n = 3) and Y/Y2/Y3 (n = 4), in addition to the previously characterized groups A (n = 7), B (n = 6), and C/C3 (n = 3). Two different plasmid patterns (Y2 and Y3) were identified from the *E*. *coli* transformants resulted from the DNA of the Senftenberg isolate BEAR101182 (Table 1). Restriction patterns of the representative plasmid from each group/subgroup are shown in Fig 2. Plasmids Y and Y2 showed nearly indistinguishable *Nla* III patterns except for an extra ~900-bp band from plasmid Y (Fig 2A); however, they were easily separated by other enzyme combinations. Digestion patterns from *Xho* I + *Eco*R V and *Xho* I + *Bgl* II are shown in Fig 2B. The diffused bands in Fig 2 were likely undigested plasmids.

All three X plasmids were in serotype Hadar isolated from turkey (ground or carcass). All group Y/Y2/Y3 plasmids were isolated from serotype Senftenberg strains. Plasmids Y2 and Y3 were isolated from colonies transformed with DNA from the same *S*. Senftenberg turkey isolate BEAR101182; the Y3 plasmid was only represented by one colony and Y2 by three colonies. Serotype Typhimurium strains (n = 4) were found to only carry group A Kan^R^ ColE1-like plasmids in this study. Three Newport strains each carried a different Kan^R^ ColE1-like plasmid group (A, B and C). Except for the four Typhimurium strains and two of the three *S*. Newport strains, all other Kan^R^ plasmid-carrying strains (~73%; n = 16) were turkey isolates including serotypes I 4,12:i,v: (n = 1), Brandenburg (n = 1), Hadar (n = 6), Newport (n = 1), Orion var. 15+ (n = 1), Schwarzengrund (n = 2), and Senftenberg (n = 4). Serotype Hadar was the most prominent serotype comprised of >27% of the strains carrying Kan^R^ ColE1-like plasmids.

### Sequencing of the *aph* gene and RNA I/II region of the novel X and Y plasmid groups

**Fig 3:**
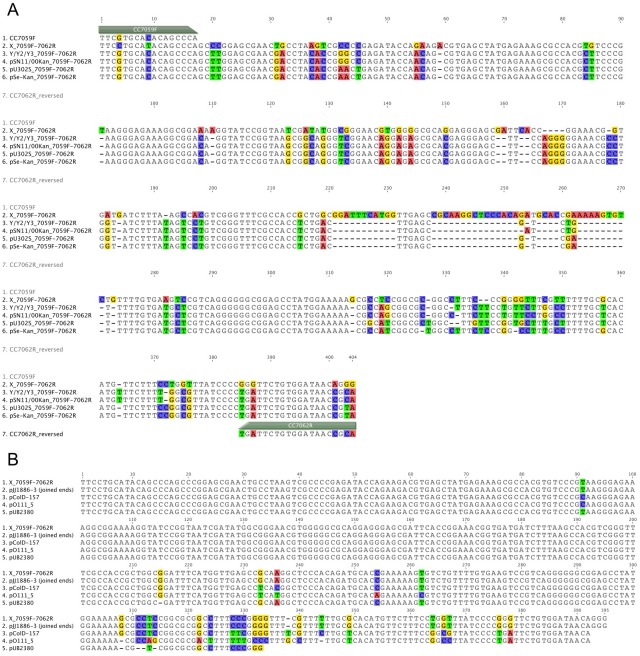
Sequence alignment of the RNA I/II regions between CC7059F and CC7062R primer binding sites. A. Alignment of representative X and Y plasmids to pU302S (group A), pSN11/00Kan (group B), and pSe-Kan (group C). B. X plasmid against top 4 BLAST hits (pJJ1886_3, pColD-157, pO111_5, and pUB2380). Non-identical nucleotides are highlighted in colors.

The *aph* gene was amplified from the plasmid DNA purified from the *E*. *coli* strain and sequenced using the primers listed in S2 Table. All *aph(3’)-I* genes from plasmids X, Y, Y2, and Y3 were identical to that of pU302S (GenBank Accession# AY333433) (data not shown). The primers CC7059F and CC7062R were used to sequence all new X and Y/Y2/Y3 plasmids directly. All Y/Y2/Y3 plasmids in this study were identical between the CC7059F and CC7062R primer binding sites, and were very similar to that of pSN11/00Kan (GQ470395; 99.1%), pU302S (AY333433; 94.3%) and pSe-Kan (HQ230976; 94.3%) (Fig 3A). Conversely, sequences of X plasmids generated from primer CC7059F showed only moderate similarities to plasmids pU302S (71.2%), pSN11/00Kan (70.2%), and pSe-Kan (71.7%) and Y/Y2/Y3 plasmids (70.5%) (Fig 3A). When this region of the X plasmids was compared against the GenBank database using BLASTN (megablast), they were found to have a sequence identity of 100% to the 5.6-kb pJJ1886-3 plasmid of a MDR *E*. *coli* isolate belonging to the ST131 *H30*-Rx sublineage (CP006787) [39], 98.5% to the kanamycin resistance plasmid pUB2380 (AJ008006; 8561 bp), and 96.7% to the 6.7-kb pColD-157 plasmid of *E*. *coli* serotype O157:H7 isolate CL40 cured (Y10412) [40] (Fig 3B). Unexpectedly, primer CC7062R did not work for sequencing of X plasmids. Upon further inspection, there were two mismatches on the X plasmids within the last three nucleotides at the 3’-end of the CC7062R primer binding site. It is unclear exactly how the primer CC7062R could have worked for screening for X plasmids, although it is possible that a small proportion of the primers could have lost the last (mismatched) nucleotide, or the original *S*. *enterica* strain carried another ColE1-like plasmid that does not encode the kanamycin resistance gene.

## Discussion

The *Salmonella* serotypes carrying Kan^R^ ColE1-like plasmids identified in this study were very different from those described in the previous study using 2005 NARMS diagnostic samples [25]. NARMS isolates from our previous study were diagnostic isolates, obtained from sick animals from diagnostic labs and the National Veterinary Lab Services. Slaughter isolates tested in the present study originated from USDA’s Hazard Analysis Critical Control Point (HACCP) sampling program of federally inspected slaughter and processing facilities across the United States. *Salmonella* serotypes and antimicrobial susceptibility in the NARMS strain collection used in this study may be biased as a result of increased sampling of both noncompliant processing facilities and facilities with serotypes commonly linked with human infections [41]. Thus the collection may be more skewed towards contaminating isolates in the facilities and not the true population of *Salmonella* in the natural environment or animal hosts.

In the present study, the Kan^R^ plasmid-carrying isolates were predominantly from turkey. The starting set of 223 kanamycin-resistant isolates were dominated by serotypes Dublin, Heidelberg, and Typhimurium, but somewhat evenly distributed among all animal source/species (20–30%). However, isolates shown to carry the Kan^R^ ColE1-like plasmids were mostly from turkey and some from cattle (ground beef). Among these Kan^R^ plasmid-carrying isolates, serotype Hadar (all six strains from the total isolates screened) made up ~27%, followed by Typhimurium and Senftenberg (four strains each). This is in strong contrast to our previous study of the 2005 NARMS diagnostic samples (~50% from cattle/dairy cattle) which showed that nearly 60% of the Kan^R^ plasmid-carrying strains were from serotype Newport, followed by Typhimurium (31.8%). In addition to changes in the NARMS isolate pool and sampling scheme (slaughter vs. diagnostic), differences in results are also likely due to the products from which they were isolated. Additional information on the differences in prevalence of *S*. *enterica* isolates from humans, retail meats, and food animals can be found in the NARMS 2011 Executive Report [42].

In the previous study of NARMS 2005 samples, strains carrying group A/A2 Kan^R^ ColE1-like plasmids comprised nearly half of the strains (45.5%), followed by group B/B2 (31.8%), and group C/C2/C3 (22.7%) [25]. The plasmid groups were more evenly distributed in the current study of NARMS 2010–2011 samples: 31.8% group A, 27.3% group B, and 13.6% each of groups C/C3, X, and Y/Y2/Y3. Plasmid groups A, B, and C/C3 were found in *S*. *enterica* isolates from different animal species (cattle and turkey) and from different years (2005, 2010/2011) and sample types (diagnostic vs. slaughter), suggesting that they may be circulating among different food animal groups, or may have an environmental niche that resulted in widespread presence over an extended time frame.

Plasmid groups X and Y appeared to be exclusively present in serotypes Hadar and Senftenberg, respectively, and all were isolated from turkey. Serotype Typhimurium predominantly carried group A plasmids: all four Typhimurium isolates carried group A plasmids in this study and six of the seven Typhimurium isolates carried A plasmids (and one carried a group C plasmid) in the NARMS 2005 collection. In this study, group B plasmids were distributed among a variety of serotypes, including Newport, Hadar, and Senftenberg, compared to Newport and Heidelberg in the 2005 study. Also, very few Newport isolates were identified in this study, whereas approx. half of the Kan^R^ ColE1-like plasmid-carrying isolates were Newport in the previous 2005 study. The differences observed in the plasmid types and serotype distribution is likely due to the differences in the isolate selection available from the two studies.

All of the Kan^R^ ColE-like plasmids carried *aph3’-I*, encoding aminoglycoside 3’- phosphatase, responsible for the kanamycin resistance phenotype. It is possible that the kanamycin resistance may be conferred by genes other than *aph3’-I*; however, we did not use PCR results of the primer pair APH-F1 and APH-R1 as a prerequisite, thus this did not bias our screen. There was a portion of the strains (10/66 = 15.2%) that appeared to carry large plasmids capable of conferring resistance to Kan. In the previous 2005 study, 30 were positive for the ColE replicon by PCR from a total of 102 Kan^R^ *S*. *enterica* isolates; 23 were capable of transforming *E*. *coli* to Kan^R^, and seven out of 30 ColE(+) strains (23%) were classified as “non-transformable” (N/T) [25], possibly due to the resistance genes on the chromosome or large plasmids. In this study, there were 20 (30%) “non-transformable” and 10 (15%) possible strains carrying large plasmids. Previously, plasmid minipreps were prepared from the *S*. *enterica* strains, while total genomic DNA was used in the current study. It is likely that DNA from the current procedure may favor large plasmid isolation, thus resulting in a higher proportion of the “smear” patterns from the *E*. *coli* transformants compared to that of the previous procedure using plasmid minipreps that is inefficient in isolating large plasmids. Differences in serotypes and animal source/species may also be responsible for the observed differences in the success rate of identifying small Kan^R^ ColE1-like plasmids.

The highly conserved region (> 99.5% identity) between X plasmid and pJJ1886-3 spanned nearly 4 kb, extending beyond the RNAI/II replication region, into *rom* and a few mobilization genes (Chen and Strobaugh, unpublished result). Plasmid pJJ1886-3 is one of five plasmids in the MDR *E*. *coli* stain JJ1886 (resistant to beta-lactams, fluoroquinolones, aminoglycosides, and chloramphenicols) isolated from a patient with fatal urosepsis [43]; however, the only plasmid harboring resistance genes in strain JJ1886 is the large 110-kb plasmid pJJ1886-5. Previous to this study, the similarities between groups A, B, and C Kan^R^ ColE1-like plasmids in the *S*. *enterica* isolates and other non-*Salmonella* plasmids (such as *E*. *coli* colicin plasmids pCol-let and pColD-157) were restricted to a smaller region (ranging from a few hundred bp to ~ 2.5-kb) and/or with moderate levels of identity (~ 80–90%) [24,26]. This is the first incidence of a Kan^R^ ColE1-like plasmid in our studies showing nearly identical sequences in such an extensive region to a plasmid from a pathogenic *E*. *coli* isolate, pointing to the possibility that the X-like plasmid was recently acquired by either *S*. *enterica* or the pathogenic *E*. *coli* isolate via a horizontal transfer event such as those previously described in the literature. Reisner and colleagues demonstrated that conjugative plasmid transfer promotes biofilm formation in co-cultures (mixed population) comprised of natural *E*. *coli* isolates [44]. Klümper and colleagues evaluated the mobilization of IncQ plasmid RSF1010 by IncP plasmid RP4 in *Pseudomonas putida* via direct mobilization (both plasmids co-reside in the same donor bacterium) and retromobilization, which requires the conjugative plasmid transfers first into the strain carrying the mobilizable plasmid and then mediates the mobilizable plasmid transfer back through the established connection; the mobilization potential of non-self-transmissible plasmids in mixed communities of different plasmid content was also quantified [45]. It is conceivable that the plasmid content of the microbial community in the natural habitat (GI track of the food animals, humans, or the environment) will have similar influence on the mobilization of these ColE1-like plasmids, with or without the co-resident conjugative plasmid. The mobilization capability of these Kan^R^ ColE1-like plasmids by conjugative plasmids, such as F and IncP, is currently being investigated in our lab. With increasing availability of next-generation whole genome sequencing data, the small plasmid database is poised to expand significantly, which will improve our ability to identify and categorize these plasmids and possibly derive their evolution.

In conclusion, Kan^R^ ColE1-like plasmids are widely distributed among different *Salmonella* serotypes in 2010–2011 NARMS isolates as in the 2005 isolates. Turkey appeared to be a major animal source for *S*. *enterica* strains carrying Kan^R^ ColE1-like plasmids in the current study, in addition to cattle. Two new plasmid groups were isolated and partially sequenced. Due to the differences in the isolate selection and selection bias, we cannot derive trend changes between the two studies. The results from this report warrant further research focusing on small plasmids harboring resistance genes.

## Supporting information

S1 Table *S*. *enterica* NARMS isolates screened in this study. (XLSX)

S2 Table Oligonucleotide primers used in this study. (DOCX)
