# Supplementary material for: Isolation and characterization of two novel groups of kanamycin-resistance ColE1-like plasmids in Salmonella enterica serotypes from food animals
Source: PLoS One. 2018 Mar 7;13(3):e0193435. doi: 10.1371/journal.pone.0193435 (PMC5841774; doi:10.1371/journal.pone.0193435)
Supplement: S2 Table — (DOCX) [file pone.0193435.s002.docx]

**S2_Table. Oligonucleotide primers used in this study.**

| **Primer name** | **Sequence (5’ to 3’)** | **Purpose** | **Reference** |
| --- | --- | --- | --- |
| CC7059F | ttcgtgcacacagccca | ColE1 typing | 25 |
| CC7062R | tgcggttatccacagaatca | ColE1 typing | 25 |
| APH-F1 | ATGAGCCATATTCAACGGG | *aph* PCR and sequencing | 25 |
| APH-R1 | AGAAAAACTCATCGAGCATC | *aph* PCR and sequencing | 25 |
| APH-R2 | CGGTGAGAATGGCAAAAGC | *aph* sequencing | 25 |
| APH-F4 | CGATTCCTGTTTGTAATTGTCC | *aph* sequencing | 25 |
